# Insights into the Mechanism of Pre-mRNA Splicing of Tiny Introns from the Genome of a Giant Ciliate *Stentor coeruleus*

**DOI:** 10.3390/ijms231810973

**Published:** 2022-09-19

**Authors:** Jirayu Nuadthaisong, Tanaporn Phetruen, Chanakan Techawisutthinan, Sittinan Chanarat

**Affiliations:** Laboratory of Molecular Cell Biology, Department of Biochemistry, Faculty of Science, Mahidol University, Bangkok 10400, Thailand

**Keywords:** *Stentor coeruleus*, pre-mRNA splicing, spliceosomes, snRNAs, tiny introns, steric-clash avoidance

## Abstract

*Stentor coeruleus* is a ciliate known for its regenerative ability. Recent genome sequencing reveals that its spliceosomal introns are exceptionally small. We wondered whether the multimegadalton spliceosome has any unique characteristics for removal of the tiny introns. First, we analyzed intron features and identified spliceosomal RNA/protein components. We found that all snRNAs are present, whereas many proteins are conserved but slightly reduced in size. Some regulators, such as Serine/Arginine-rich proteins, are noticeably undetected. Interestingly, while most parts of spliceosomal proteins, including Prp8′s positively charged catalytic cavity, are conserved, regions of branching factors projecting to the active site are not. We conjecture that steric-clash avoidance between spliceosomal proteins and a sharply looped lariat might occur, and splicing regulation may differ from other species.

## 1. Introduction

*Stentor coeruleus* is a giant single-celled ciliate which lives in freshwater environments worldwide. The organism is relatively large (up to 2 mm in length) and has a clear anterior–posterior axis, detailed cortical patterning, and an ability to repair itself even after being damaged with large wounds in the plasma membrane [1,2]. These unique characteristics of *S. coeruleus* make it an excellent model organism, and it has thus long been used as a model organism for studying unicellular regeneration, wound response, and cell repair mechanisms [3,4]. Although the organism has been studied by many scientists for several decades, its genome and transcriptome have just recently been sequenced [4,5]. Since the genome sequencing reveals that *Stentor* uses the standard genetic code, unlike many ciliates, it has been proposed that the protist may have branched from others before ciliate-specific genetic codes arose [4]. Moreover, despite its large cell size, spliceosomal introns are extremely tiny—merely 15 to 16 nucleotides (nt) long. Given that the median intron length in protein-coding genes of budding yeast *Saccharomyces cerevisiae* and humans is approximately 148 and >1300 nucleotides, respectively [6,7], the exceptionally small introns of *S. coeruleus* raise an important question about whether the protist may have an unusual mechanism of pre-mRNA splicing [2].

Pre-mRNA splicing is an RNA processing step which removes non-coding introns from a premature transcript [8,9,10]. The process is catalyzed by a megadalton ribonucleoprotein complex of spliceosome, which is involved with five small nuclear ribonucleoproteins (snRNPs)—U1, U2, U4, U5 and U6—and many non-snRNP-associated proteins [9,10,11]. To function, the spliceosome has to be de novo-assembled on each intron in a step-wise manner [9,10,11]. First, U1 and U2 snRNPs recognize the 5′ splice site (SS) and branchpoint (BP) sequence of the intron, respectively, to form a pre-spliceosomal complex. The pre-assembled U4/U6.U5 tri-snRNP then joins to shape a pre-catalytic spliceosome. Subsequently, an extensive structural rearrangement of both protein and snRNA components of the spliceosome occurs to activate the snRNPs [9,10,12]. Upon the activation, the spliceosome coordinates the two transesterification reactions required for intron removal and the completion of the splicing cycle [9,10,11,13]. For proper splicing fidelity, spliceosome assembly and activation are tightly controlled by several ATP-dependent RNA helicases as well as base-pairing interactions between snRNAs of the spliceosome and the intronic sequences of the pre-mRNA [14,15].

Given that the remarkably small introns of *S. coeruleus* are recognized and spliced out by the relatively large spliceosome, we wondered how splicing in this species is regulated and therefore analyzed intronic sequences and spliceosomal components at the genomic level. Here, we show several intriguing features of *Stentor* introns. Moreover, we informatically identify snRNA and protein components of the *Stentor* spliceosome. We also propose a base-pairing scheme of the spliceosomal active site and its interaction with the intronic substrate. Intriguingly, although most spliceosomal protein homologs are present and similar to their vertebrate counterparts, the size of most spliceosomal proteins is reduced and certain regions of branching factors at the active site are non-conserved. We conjecture that an avoidance of steric clashes between spliceosomal components and a looped structure of intron lariat may take place in this species and hypothesize that the regulation of pre-mRNA splicing of *Stentor* introns may be distinct to others due to its ordinarily small size of intron.

## 2. Results and Discussion

### 2.1. Features of Stentor Introns

**Figure 1:**
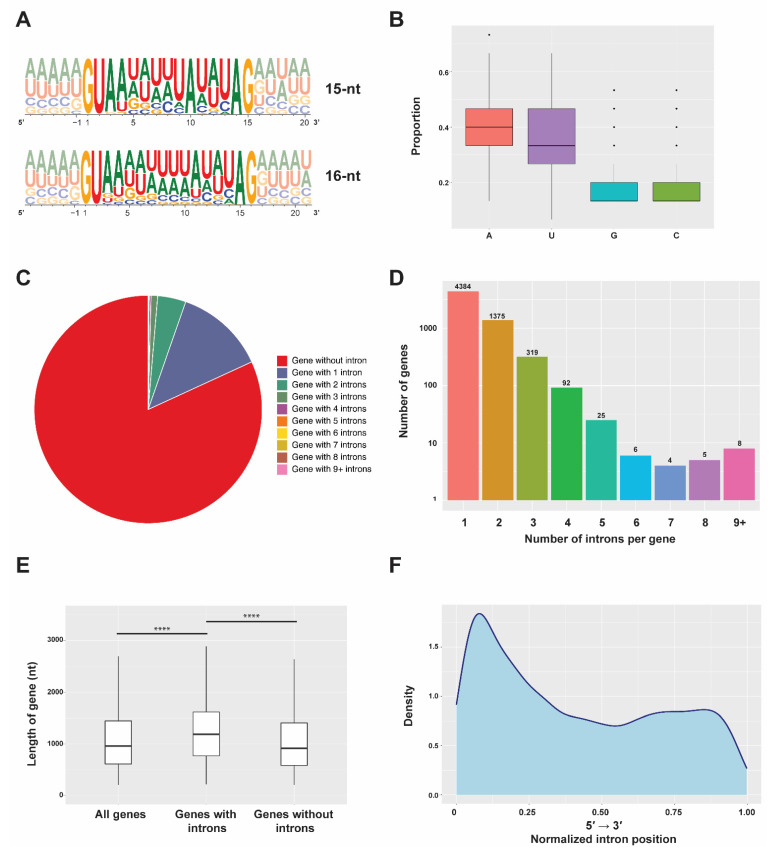
Features of *Stentor* introns. (**A**) Sequence conservation of nucleotides (sequence logos) of all annotated introns of *S. coeruleus*. Introns 15 and 16 nucleotides in length were compared. Height of the letters depicts the relative frequency of each nucleotide in individual positions. Partial sequences of 5′ and 3′ exons are shown in gray. (**B**) Proportions of nucleotide bases of each intron were plotted as a box plot. (**C**,**D**) Frequency of genes with different numbers of introns per gene in the genome of *S. coeruleus*. Number above each bar indicates the number of genes. (**E**) Length of all genes; genes with or without introns in *S. coeruleus* were plotted as a box plot. nt, nucleotides. (**** *p* < 0.0001) (**F**) Graphic representation of the location of the *Stentor* introns within the gene body. Each location is represented as the ratio of intron start location to gene length.

To gain more insight into the splicing mechanism in *Stentor coeruleus*, we first analyzed features of intronic sequences using reported genomes and databases [4,16]. In this species, 8806 introns were annotated [4,16]. Among these, 8173 of them (92.81%) were 15 nucleotides (nt) long, while the rest (633 intronic sequences; 7.19%) were 16 nt long. From the annotation general feature format (GFF) and the genomic sequence of the ciliate, we then extracted the sequences of each annotated intron to analyze it in further detail. According to the frequency plots [17], most of the introns start with GU and end with AG nucleotides, most commonly observed 5′-splice sites (SS) and 3′-SS of introns of major spliceosome in many eukaryotic species. Though the branchpoint region of *Stentor* introns does not show a strong consensus, the majority of the branchpoint adenosine (BP-A) of 15 nt-long and 16 nt-long introns almost unvaryingly reside in the 10th (8005 introns; 90.90% of all introns) and 11th position (442 introns; 5.02% of all introns), respectively (Figure 1A). On the other hand, approximately 2% of introns in each case (168 introns or 1.91% for 15 nt-long sequences and 191 introns or 2.17% for 16 nt-long introns) harbor the BP-A at other positions. Strikingly, introns of the ciliates are enriched with adenine (A) and uracil (U), as the sum of the percentage of both nucleotides (AU content) is as high as 75.63% (Figure 1B). This analysis suggests that splicing of most *Stentor* introns, when spliced, would result in an AU-rich 10 to 11 nt-long circular lariat with a 5 nt-long 3′ tail. It is interesting to note that *Stentor*’s 5′ exon seems to be unlike that of mammals, in which the last nucleotide of the exon bordering the splice donor site is usually a G (Figure 1A).

Next, we analyzed global features in *Stentor* introns in biological contexts. We first simply asked how abundant intronic sequences are in *S. coeruleus*. We found that while a larger number of 28,064 genes lack introns, 6218 genes have the sequences, indicating that only 18.14% of genes contain introns (Figure 1C). Among the intron-containing genes, most of them have merely one intron, while a large proportion seems to have only a few (Figure 1C,D). Though some genes harbor more than eight introns, we were uncertain whether all of them are functional and actually spliced; further in vivo experiments must be required. Interestingly, we observed that the presence of introns is correlated with a longer gene length; the median gene length of intron-containing genes is 1230 nucleotides and significantly longer than that of intron-less genes, which is only 939 nucleotides in length (Figure 1E). Moreover, we also observed that introns have a positional bias toward the 5′ end of each gene (Figure 1F); a similar phenomenon has also been found in many eukaryotic species, including *Saccharomyces cerevisiae* [18,19]. Gene Ontology (GO) and pathway enrichment analyses of genes harboring introns showed significant enrichments of genes involving catalytic activity, ion binding, organic cyclic compound binding, and several metabolic processes, suggesting potential physiological roles of gene regulation at the level of pre-mRNA splicing in *S. coeruleus* (Figures S1 and S2 and Table S1).

### 2.2. Identification of Spliceosomal snRNAs in S. coeruleus

**Figure 2:**
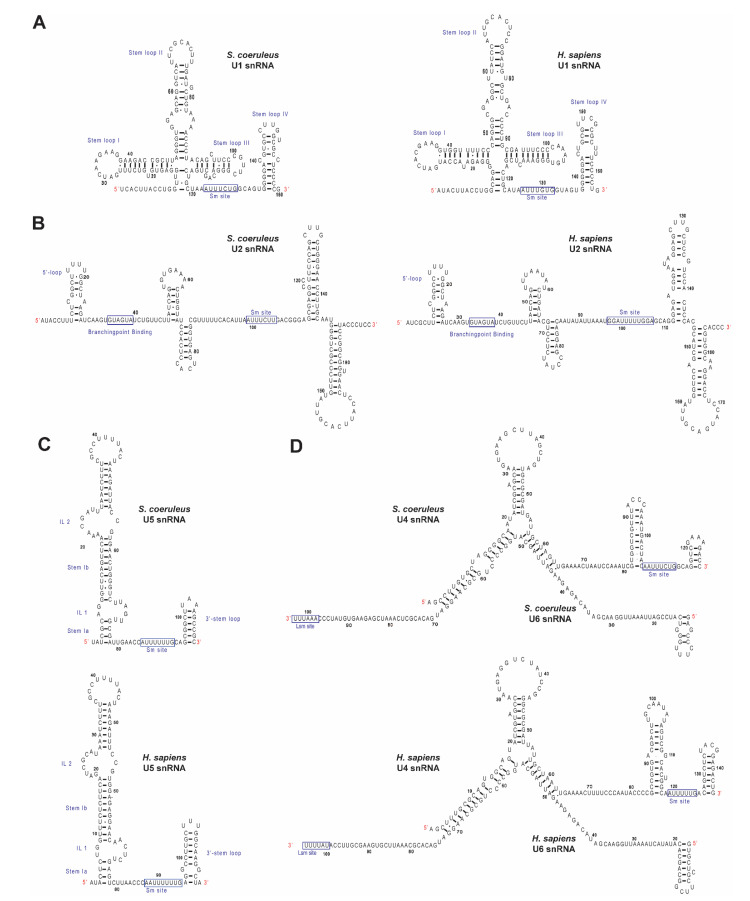
Sequences and predicted secondary structures of *S. coeruleus* spliceosomal snRNAs in comparison with their human counterparts. Predicted secondary structures of U1 (**A**), U2 (**B**), U5 (**C**), and U4/U6 (**D**) snRNAs are shown. Nucleotides are numbered from 5′ to 3′, and putative Sm/Lsm/Branchpoint binding sites are boxed. Conserved loops are indicated in roman numerals.

Though the introns of *S. coeruleus* are exceptionally small and may require a unique spliceosomal regulation, little is reported about splicing machineries in the ciliate. Thus, we next aimed at identifying all components of its spliceosome, including U-snRNAs and associated proteins. Since the introns of the protist harbor conventional GU-AG motives (Figure 1A), we speculated that all major spliceosomal snRNAs might be present. As searching the U-snRNA candidates based on primary DNA sequence similarity often fails due to the low sequence similarity, we used sequences of U-snRNAs from the Rfam database [20] to seek the corresponding U-snRNAs from the *S. coeruleus* genome using the cmbuild and cmsearch programs of the Infernal package [21]. As expected, we found all snRNAs of the major spliceosome (Figure S3A–E). Comparisons of sequences, the Sm/Lsm binding site, covariance model, and secondary structure showed that all predicted U-snRNAs of *S. coeruleus* are similar to other eukaryotic snRNA counterparts (Figure 2A–D). It is interesting to note that none of snRNAs of the minor spliceosome—U11, U12, U4atac, and U6atac—were found using the above strategy. Additionally, as it is consistent with the notion that the existence of the U12-type introns has not been reported, we conjecture that the primary events of pre-mRNA splicing in *S. coeruleus* are involved with the major spliceosome and the U2-type introns.

Intrigued by the above findings, we further analyzed unique features of all five spliceosomal U-snRNAs. First, *Stentor* U1 snRNA exhibits a conserved region, ACUUACCU, that potentially binds to the 5′ SS of introns, as we found the sequence identical to that of the Rfam model of U1 snRNA (Figure 2A). We also observed that the branchpoint-binding motif GUAGUA in the predicted U2 snRNA of *Stentor* is also highly conserved (Figure 2B), suggesting that intron recognition mechanism may be similar to that of other spliceosomes. Since the sequence of putative U4 snRNA of *S. coeruleus* could be very complementary with the sequence of putative U6 snRNA (Figure 2D) and the sequence and secondary structure U5 snRNA is highly conserved (Figure 2C), we conjecture that the formation of the snRNA backbone of the U4/U6.U5 tri-snRNP may be indistinct to that of other species. From these findings, we conclude that the spliceosome of *S. coeruleus* contains all five snRNAs, the sequences and features of which are most likely similar to their homologs in other eukaryotes.

### 2.3. Identification of Protein Components of Spliceosomal snRNPs in S. coeruleus

Next, we asked whether spliceosomal proteins are also conserved in *S. coeruleus*. To this end, we first obtained information of each protein from the Uniprot database and used the Uniprot proteome gene identifier (ID) as query in the protein Basic Local Alignment and Search Tool (BLASTP) against the non-redundant protein sequences (nr) database with an Expect I-value cut-off of 1 × 10^−5^. Because the assembly of the *S. coeruleus* genome is yet to be completed [4] and the proteome database of annotated proteins may still lack certain sequences, we employed the translated nucleotide BLAST (TBLASTN) operation mode against the whole-genome shotgun contigs (wgs) of *S. coeruleus* with an E-value cut-off of 1 × 10^−5^ if the initial BLASTP failed to identify any significant hit (Table 1). First, we analyzed Sm and Sm-like (Lsm) proteins, the core proteins that associate with the U1, U2, U4, and U5 snRNAs and the U6 snRNA, respectively. Consistent with the presence of all five U-snRNAs and the conserved Sm/Lsm binding sites (Figure 2), all seven Sm (Sm B, D1, D2, D3, E, F, and G) and seven Lsm (Lsm2 to Lsm8) proteins were identified by BLAST (Table 1), suggesting that Sm/Lsm hetero-heptameric ring complexes are most likely formed and possibly interact with the corresponding U-snRNAs as in other eukaryotes.

We next investigated whether U1, U2, and U4/U6.U5 tri-snRNP specific spliceosomal proteins are present in *S. coeruleus*. For the U1 snRNP, our BLAST analysis showed that Nam8/TIA1, Prp39/PRPF39, and all three core U1-specific proteins—Mud1/SNRPA (U1A), Yhc1/SNRPC (U1C), and Snp1/SNRNP70 (U1-70k)—are conserved, while the more peripheral U1 snRNP components are undetected by either BLASTP or TBLASTN (Table 1). Strikingly, however, all protein components of U2-snRNP- and U2-snRNP-associated complexes were identified except U2SURP. Since the sequences and predicted secondary structures of U1 and U2 snRNAs are conserved (Figure 2) and the two undiscoverable proteins are likely vertebrate-specific factors CHERP and U2SURP (Table 1) [22], we conjecture that the core complexes of U1 and U2 snRNPs as well as their associated factors are plausibly similar to those of other eukaryotic species. We subsequently investigated the presence of tri-snRNP proteins at the genomic level. Out of 18 proteins, all except 3 were identified by BLAST (Table 1), suggesting that U4/U6.U5 tri-snRNPs of *S. coeruleus* and those of others may share similar structures and functions. We conclude from our findings that all five snRNP complexes of *S. coeruleus* may be formed and function in a similar manner to the complexes in other species.

### 2.4. Identification of Stentor Spliceosomal RNA Helicases and Other Non-snRNP Proteins Involving Spliceosome Assembly and Activation

Pre-mRNA splicing involves multistep assembly and activation of the spliceosome. During the early step of spliceosome assembly, U1 and U2 snRNPs function by recognizing the intronic sequences of a pre-mRNA and forming a pre-spliceosomal complex known as the A complex [9,10]. Subsequently, the pre-assembled U4/U6.U5 tri-snRNP joins and forms the pre-catalytic spliceosome (B complex), which then undergoes ATP-dependent conformational rearrangement of its protein and snRNA components [9,10]. Remodeling of the B complex by the RNA-dependent helicase Brr2/SNRNP200 results in dissociation of the U1 and U4 snRNP complexes and thereafter the recruitment of several non-snRNP proteins, including the NineTeen Complex (NTC) and NTC-related proteins, to form the activated spliceosome (B^act^ complex) [9,10,23]. After further structural changes by the ATP-dependent RNA helicases Prp2/DHX16 and Prp16/DHX38 and dynamic association/dissociation of proteins, the catalytic spliceosome (C complex) is subsequently formed [9,10,14,15].

Given that spliceosome assembly and activation are highly dynamic and important for intron recognition, exon–intron arrangement, and the removal of introns, we next focused on identification of the spliceosomal proteins that are involved in these steps. First, we observed that all seven spliceosomal ATP-dependent RNA helicases—Prp2/DHX16, Prp5/DDX46, Prp16/DHX38, Prp22/DHX8, Prp28/DDX23, Prp43/DHX15, and Brr2/SNRNP200—and one GTPase Snu114/EFTUD2 were all identified in the *S. coeruleus* genome, implying that the ciliate may also utilize ATP and GTP during spliceosome assembly and activation steps (Table 1). Although the protist seems to lack certain components of splicing complexes, such as proteins recruited during the A complex stage, the NTC and NTC-related proteins, the C complex, and step II proteins, almost all proteins recruited at the B and B^act^ complexes stage are present (Table 1).

Intriguingly, we observed that while orthologs of heterogeneous nuclear ribonucleoproteins (hnRNPs) were identified, all Serine/Arginine (SR)-rich splicing factors and SR-related proteins were absolutely absent from our search results. Both hnRNPs and SR-family proteins function as general splicing repressors and activators, respectively [24,25,26,27]. Mechanistically, they interact with cis-elements in the transcripts and then recruit and/or stabilize components of the core spliceosome [26,28]. The lack of SR and SR-related proteins may be because *S. coeruleus* does not need to selectively promote the removal of specific introns. Additionally, exon skipping (ES) may not occur in the species. This may also be explained by the fact the size of *Stentor* introns is mostly constant at 15-16 nucleotides long [4] (Figure 1A); if the ciliate is able to skip an exon, which is a long nucleotide stretch of nucleotides, the size of its introns must be more deviated. The presence of hnRNPs, on the other hand, indicates that the repression of intron splicing may occur in this species. This could be a splicing-mediated mechanism to alter gene isoforms, thereby controlling gene expression in the ciliate. However, given that many known hnRNPs support a broad range of non-splicing biological functions—including mRNA stabilization and nuclear export and transcriptional and translational regulations [29,30,31,32]—we were uncertain whether the hnRNP orthologs found in *S. coeruleus* exclusively function in pre-mRNA splicing. Nevertheless, the absence of SR and SR-related proteins and the presence of hnRNPs may reflect the unique regulation of tiny-intron splicing and RNA metabolism as well as the relatively intron-poor nature of the protist.

It is important to note that a limitation of our work may arise as a consequence of BLAST analysis, which could fail to detect protein factors with distant homology. Additionally, because we used known splicing factors as query sequences to seek *Stentor* orthologs, species-specific splicing factors, which may also exist and contribute to the splicing of the exceptionally tiny introns in the protist, could be simply overlooked. Therefore, in order to ascertain spliceosomal components of the ciliate, proteomic and biochemical analyses are definitely required. Nevertheless, our above findings suggest that not only spliceosomal snRNA and protein components are vastly conserved in *Stentor*, but also many non-snRNP proteins are present. Our findings also suggest that the assembly and activation of the *Stentor* spliceosome might be conserved to a certain extent, but additional species-specific regulations—if any—could also take place.

### 2.5. A Model of RNA–RNA Interaction Network in Stentor Spliceosomal Active Site

In the fully assembled spliceosome, U2 and U6 snRNAs extensively base-pair with each other and help position the two reacting groups in the first step of splicing—the 5′ SS and the branchpoint region—by base-pairing with the two sequences [9,10]. The base pairing between the U2 snRNA and the branchpoint region protrudes the BP-A out from the RNA duplex [9,11]. The 2′ OH of the BP-A then undergoes a nucleophilic attack on the 5′ SS, and thereby the 5′ linkage between the BP-A and the first guanine nucleotide of the intron is formed. During the reaction, the 5′ exon is unconnected with the intron but still remains held in the active site via interactions with the U5 snRNA and associated proteins [9,10,11,15]. Next, the second step of splicing involves a nucleophilic attack by the 3′ OH group of the 5′ exon on the phosphodiester bond at the 3′ SS. Ultimately, the spliceosome dissembles and releases the lariat intron [9,11].

**Figure 3:**
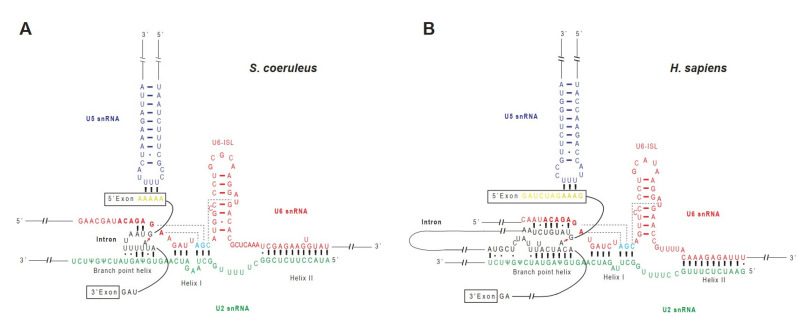
Comparison of schematic representations of active sites of the *Stentor* and human spliceosomes and the interactions with pre-mRNA substrates. (**A**) A proposed model of the RNA interaction network before the first trans-esterification reaction. The U6 snRNA (red) forms an intramolecular stem loop (ISL) and the two helices (I and II) with the U2 snRNA (green). Catalytic triad (AGC; cyan) forms three consecutive triple base-pairs with the catalytic triplex of U2 snRNA nucleotides. The nucleotides of 5′ splice site (SS) of the intron are base-paired with the ACAGAGA box of the U6 snRNA (bold), and the nucleotides of branchpoint region are base-paired with the U2 snRNA and allow the branchpoint adenosine to bulge out from the branch helix. The 5′ exon is colored in yellow, U5 snRNA in blue, and other parts of pre-mRNA in black. (**B**) Similar to Figure 3A, the previously proposed RNA interaction network of human spliceosome is shown as a reference [11].

Given that introns of *Stentor* are exceptionally small, we next asked how the U2 and U6 snRNAs base-pair with each other and with the intronic sequences to position the 5′ SS and the BP-A. To this end, we analyzed the sequences of the relevant snRNAs of *S. coeruleus*, predicted the RNA–RNA interaction network, and compared it with that of the human spliceosome (Figure 3A,B and Figure S4). The active site of the human spliceosome is formed during the transition of the B complex to the B^act^ complex and stays unchanged during the two-step transesterification reactions [9,10,11]. In the catalytically active spliceosome, the U6 snRNA forms an intramolecular stem-loop (ISL) structure and helices I and II with the U2 snRNA [11] (Figure 3B). Although the sequences of *Stentor* U2 and U6 snRNAs responsible for the formation of U6-ISL, helices I and II are slightly deviated from human sequences (Figure 3A,B and Figure S1), secondary structure and base pair predictions suggest that *Stentor* snRNAs may also form the ISL and two helices as well (Figure 3A). Moreover, the backbone nucleotides of the U6 catalytic triad (A48, G49, and C50) as well as the three nucleotides that form three consecutive triple base pairs with the triad (A41, G42, and U69) are invariantly conserved in *Stentor* (Figure 3A), suggesting that the folded RNA structure formed by the stacking of the three pairs of nucleotides might be present, too [11]. To position the pre-mRNA substrate in the active site of human spliceosome, the 5′-end region of the intron needs to be positioned by base pairing with the ACAGAGA box of the U6 snRNA and with the loop 1 of the U5 snRNA, which holds the 5′ exon (Figure 3B) [9,11]. Likewise, the branchpoint sequence of the intron also pairs with U2 snRNA to form a branch helix with the bulged BP-A (Figure 3B) [9,10,11]. In *S. coeruleus*, the ACAGAGA sequence of the U6 snRNA and the branchpoint recognition site of the U2 snRNA are highly conserved, implying that the mechanism of 5′ SS and branchpoint recognition might also occur in a similar fashion to that of other species (Figure 3A).

Next, we asked how the base paring between the pre-mRNA and U2/U6.U5 snRNAs would form. To this end, we selected the most abundant intronic sequence, GUAAUUUUUAUAUAG, as a representative (127 occurrences in 8173 introns or 1.55%; where A represents the putative BP-A) and predicted the RNA interaction network. Since the first three intron nucleotides (GUA) are stringently conserved in *Stentor* (Figure 1A), the sequence is likely able to form Watson–Crick base pairs with the U6 snRNA ACAGAGA box (Figure 3A). The branchpoint region, on the other hand, is enriched with U nucleotide—the nucleotide which potentially forms not only a Watson–Crick U-A pair, but also a wobble U-G as well as a non-canonical U•U base pair ubiquitously found in non-coding RNAs [33]. Though further validation by genetic and biochemical experiments are required, our observation suggests that the conserved branchpoint recognition site of the U2 snRNA of *S. coeruleus* possibly base-pairs with the U-rich sequence of the intron branchpoint region (Figure 3A).

**Figure 4:**
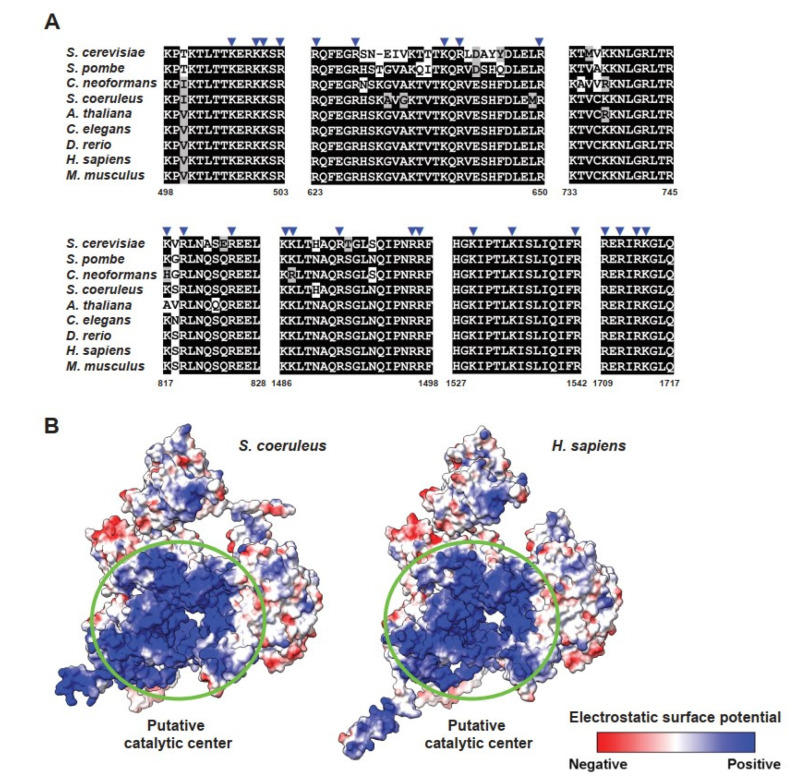
Catalytic cavity on putative Prp8 of *S. coeruleus*. (**A**) The positively charged amino acids in the catalytic activity of Prp8 are highly conserved among *S. cerevisiae*, *S. pombe*, *C. neoformans*, *A. thaliana*, *C. elegans*, *D. rerio*, *H. sapiens*, *M. musculus*, and *S. coeruleus*. Sequence alignment of the relevant regions of Prp8 homologs are shown. Positively charged amino acids that form a bordering line at the catalytic cavity of Prp8 are indicated by blue arrows. Numbers below the alignments indicate amino acid positions in the putative Prp8 protein of *S. coeruleus*. (**B**) Identification of the catalytic cavity in Prp8 by electrostatic surface potential. Human Prp8′s catalytic cavity is shown in the left panel as a reference.

The presence of all snRNAs and most of the core snRNP and non-snRNP proteins suggests that, to a certain degree, the regulation of pre-mRNA splicing in *S. coeruleus* might be conserved. Besides the network interactions between pre-mRNA and spliceosomal snRNAs, it has been demonstrated that spliceosomal proteins also play roles at the active site [9,11]. Particularly, the largest and highly conserved spliceosomal protein Prp8 occupies the central position in the catalytic core of the spliceosome [13]. We observed that the *S. coeruleus* Prp8 protein is 73.91% identical to the human homolog and the positively charged amino acids in the catalytic cavity of Prp8 share an even higher sequence identity of 94.69% with that of humans (Figure 4A). The positively charged cavity of Prp8 at the spliceosomal active site is important because it is where the RNA triplex of U2 and U6 snRNAs and the intron lariat is located [11,13]. Strikingly, analysis of the electrostatic surface potential of the cavity showed a notable similarity between the catalytic cavities of *Stentor* and human spliceosomes (Figure 4B). Taken together, we conjecture that the active site of *Stentor* spliceosome is most likely structurally and functionally similar to that of humans.

### 2.6. Regions of Branching Factors Projecting to the Spliceosomal Active Site May Be Unique in Stentor

**Figure 5:**
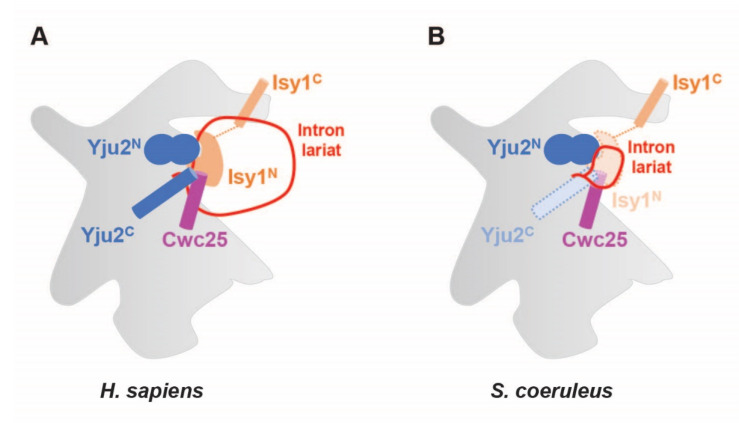
Schematic representation shows how the partial non-conservation of three branching factors Yju2, Cwc25, and Ntc30 may help prevent a steric clash with a looped intron lariat. Human (**A**) and Stentor (**B**) spliceosomes are compared.

Structural analyses of human and yeast spliceosomes reveal that protein components of the RNP enzyme are located on the surface of one side of the splicing active site; this leaves the other side freely accessible to the pre-mRNA molecule harboring introns with a vast range of lengths [13]. Our findings suggest that the spliceosome of *S. coeruleus* might be structurally and functionally similar to the spliceosome of humans. However, given that the size of the protist introns is much smaller and thus the RNA lariat might form a sharp turn of 10 nt that potentially causes a steric clash with adjacent spliceosomal proteins, we wondered how the intron would fit at the active site. To this end, we focused on the three branching factors—Yju2/CCDC94, Cwc25/CCDC49, and Ntc30/ISY1—which are adjacent to the branch region and stabilize the docking of the U2/U6 branch helix [13]. While having slightly smaller homologs than other proteins (Figure S5), the N-terminal domain of the ciliate Yju2/CCDC94, which is essential for viability and promotes branching, was highly conserved (Figure S6A). By contrast, while the N-terminal helix and three invariant tryptophan residues of Cwc25/CCDC49 (Trp^12^, Trp^24^, and Trp^72^ in CCDC49) are highly conserved in the protist, its N-terminal plug is uniquely distinct (Figure S6B). In the human spliceosome, the conserved plug with a glycine-rich motif (Gly^2^-Gly^3^-Gly^4^ in CCDC49) is located at the active site and penetrates a small cleft formed by the U2/branchpoint duplex and the helix I of the U2/U6 duplex [11,13]. Interestingly, the Cwc25/CCDC94 protein of *S. coeruleus* strikingly lacks such a conserved motif (Figure S6B). Moreover, the N-terminus of Ntc30/ISY1, which is projected into the active site of the spliceosome and forms contacts with the phosphate backbone of the intron to promote branching in other eukaryotes, is strikingly non-conserved in *Stentor* (Figure S6C). Though we are uncertain how the active site of the *S. coeruleus* spliceosome is three-dimensionally formed, the differences in these branching factors might directly and/or indirectly help avoid a steric clash with a looped structure of RNA and contribute to the formation of lariat and branching of the tiny intron of the protist (Figure 5).

## 3. Materials and Methods

### 3.1. Computational Analyses of Features of Introns of S. coeruleus

*S. coeruleus* genome data were downloaded from the *Stentor* Genome Database at http://stentor.ciliate.org/ (accessed on 22 April 2020). [4,16]. Intronic sequences were extracted from the assembled genome using coordinates obtained from the general feature format (GFF) file [16] and bioinformatics tools in the Galaxy platform [34]. Sequence logos of intronic sequences were created using WebLogo [17]. Intronic features were computed and plotted using R Studio [35]. Gene Ontology (GO) IDs of genes containing an intron in *S. coeruleus* were retrieved from UniProt. The GO analysis was run on g:Profiler [36] using *Tetrahymena thermophila* and *Paramecium tetraurelia*, members of the phylum Ciliophora, as *S. coeruleus*, an organism input parameter. The top three enriched GO IDs in the molecular function (MF), biological process (BP) and cellular component (CP) were listed. To compare sequences of genes harboring introns at the genomic and transcriptomic levels, genomic DNA and mRNA sequences were retrieved from StentorDB [16] and recently published RNA sequencing experiments performed with *S. coeruleus* [5], respectively. Multiple alignments were performed using Clustal Omega with default settings and visualized with the pyBoxShade program [37,38]. Statistical analysis was performed using two-tailed unpaired Student’s t-test in GraphPad Prism 9 software [39].

### 3.2. Identification of Spliceosomal Proteins in the S. coeruleus Genome

To identify protein components of the spliceosome in the *S. coeruleus* genome, the information of each spliceosomal protein was obtained from the Uniprot database [40]. Human spliceosomal proteins (listed in Table 1) were employed as queries in batch in a protein Basic Local Alignment and Search Tool (BLASTP) against the non-redundant (nr) database for *S. coeruleus* protein sequences on the National Center for Biotechnology Information (NCBI) website with an Expect (E)-value cut-off of 1 × 10^−5^ [41]. For proteins with no ortholog detected by the BLASTP search, the translated nucleotide BLAST (TBLASTN) operation mode was employed against whole-genome shotgun contigs (wgs) of *S.* *coeruleus* with an E-value cut-off of 1 × 10^−5^ [41]. We used yeast-specific spliceosomal proteins as queries instead when the information of human orthologs was unavailable. Multiple alignments were performed using Clustal Omega with default settings and visualized with the pyBoxShade program [37,38].

The putative catalytic center of *Stentor* Prp8 was predicted by HHpred in conjunction with MODELLER tools [42,43]. Structural comparison and electrostatic surface potential were carried out using UCSF ChimeraX Daily Build version (version 1.3; 7 September 2021) [44].

### 3.3. Prediction of S. coeruleus U snRNA Candidates

The Infernal package was downloaded from http://eddylab.org/infernal/ (access on 13 April 2020) [21]. Alignments of all U snRNAs of both major and minor spliceosomes were downloaded from Rfam (U1, Rfam: RF00003; U2, Rfam: RF00004; U4, Rfam: RF00015; U5, Rfam: RF00020; U6, Rfam: RF00026; U11, Rfam: RF00548; U12, Rfam: RF00007; U4atac, Rfam: RF00618; U6atac, Rfam: RF00619) [20]. Covariance models (CMs) of the RNAs were built using the ‘cmbuild’ program in the Infernal package. Then, the spliceosomal snRNAs of *S. coeruleus* were identified by the Infernal ‘cmsearch’ program against the assembled sequences of a reference genome for the *S. coeruleus* [4,16]. Each result from the ‘cmsearch’ program consisted an alignment and a score, all of which were required to be above zero to be considered as a hit [21]. All Infernal programs were run under the Linux operative system with default settings [21].

## 4. Conclusions

**Figure 6:**
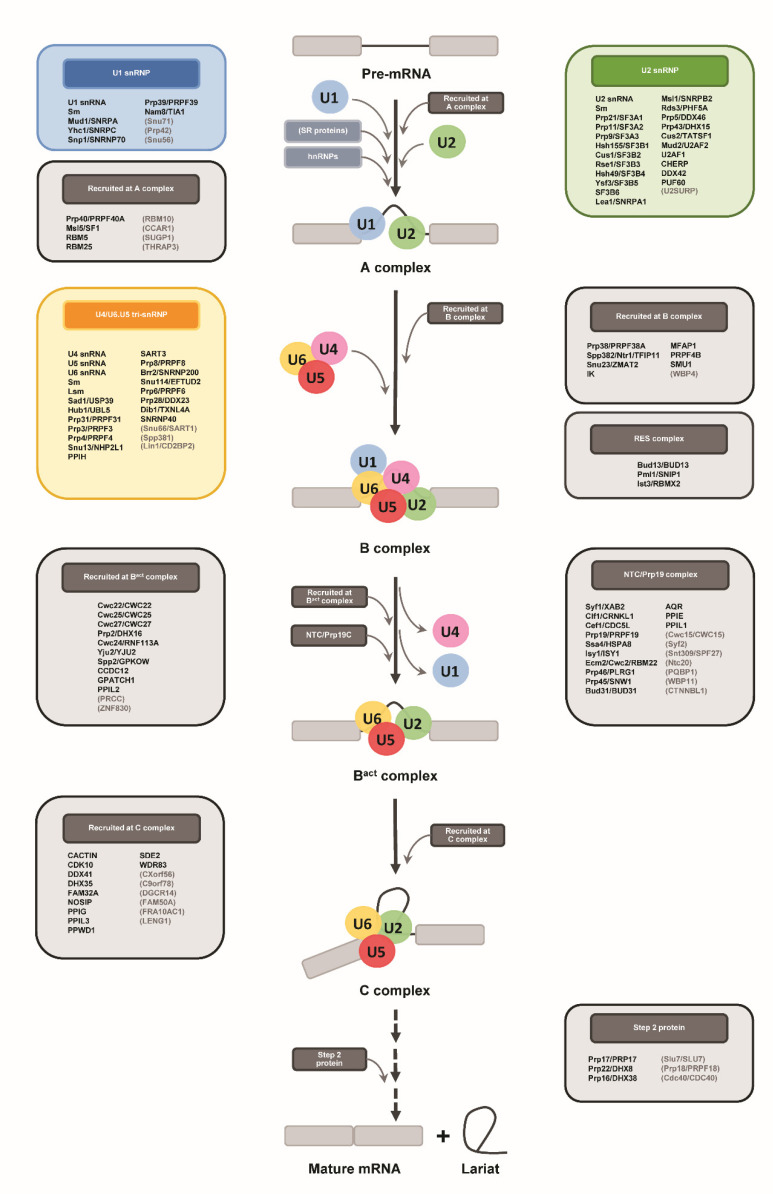
Schematic representation shows spliceosomal proteins which are conserved in *S. coeruleus*. Genes that are found in genome of the ciliate are written in bold, those unidentified in brackets.

In this study, we analyzed features of introns of *S. coeruleus* and identified snRNA and protein components of its spliceosome (Figure 6). We also propose a base paring model of the spliceosomal active site and discuss its association with an intron sequence. Although most spliceosomal proteins were conserved in the ciliate, their size is reduced. Moreover, the regions of certain branching factors that are adjacent to the spliceosome active site are noticeably non-conserved, suggesting its unique mechanism of active-site arrangement possibly for the avoidance of steric clashes between the intron lariat and spliceosomal components. Though there are limitations in our computational approach and further genetic and biochemical analyses are required, our findings provide an insight into splicing of tiny introns of *S. coeruleus*.

To date, it is unclear what environmental and/or intrinsic factors cause such a reduction in introns in the ciliate. Additionally, there are still open questions regarding whether vertebrates, including humans, could splice such a small intron, and if not, what the smallest size of the intronic sequences could be. Since small introns are unusual in the human genome and most likely overlooked, the capability of the splicing—either constitutively or stress-induced—could potentially increase mRNA isoforms and thereby the diversity of proteins, some of which might be implicated in the development of human diseases. These intriguing possibilities remain to be explored.

## Figures and Tables

**Table 1:** Yeast and human spliceosomal proteins and *S. coeruleus* orthologs identified by BLAST algorithm.

Protein/Complex	*S. cerevisiae* ^a^	*H. sapiens* ^a^	*S. coeruleus* ^b^				BLAST Operation Mode ^c^
			Accession No.	Query Coverage	E-Value	% Identity	
Sm proteins	SmB	SNRPB	OMJ80606.1	48%	2 × 10^−13^	35.42%	B
	SmD1	SNRPD1	OMJ74037.1	75%	3 × 10^−13^	40.00%	B
	SmD2	SNRPD2	OMJ84976.1	79%	2 × 10^−29^	55.91%	B
	SmD3	SNRPD3	OMJ79831.1	81%	2 × 10^−30^	50.00%	B
	SmE	SNRPE	OMJ89702.1	93%	3 × 10^−21^	39.77%	B
	SmF	SNRPF	OMJ90477.1	84%	2 × 10^−20^	50.68%	B
	SmG	SNRPG	OMJ87882.1	94%	1 × 10^−14^	43.84%	B
Lsm proteins	Lsm2	Lsm2	MPUH01000040.1	94%	1 × 10^−8^	31.52%	T
	Lsm3	Lsm3	OMJ80606.1	75%	9 × 10^−6^	33.80%	B
	Lsm4	Lsm4	MPUH01000270.1	54%	2 × 10^−16^	40.79%	T
	Lsm5	Lsm5	OMJ92196.1	69%	2 × 10^−5^	35.82%	B
	Lsm6	Lsm6	MPUH01000102.1	87%	1 × 10^−13^	41.43%	T
	Lsm7	Lsm7	OMJ69462.1	68%	2 × 10^−11^	37.97%	B
	Lsm8	Lsm8	OMJ73124.1	60%	1 × 10^−9^	34.29%	B
U1 snRNP	Mud1	SNRPA	OMJ89879.1	32%	5 × 10^−28^	49.46%	B
	Yhc1	SNRPC	OMJ82811.1	38%	7 × 10^−15^	48.39%	B
	Snp1	SNRNP70	OMJ72809.1	65%	6 × 10^−24^	33.50%	B
	Prp39	PRPF39	OMJ71629.1	66%	6 × 10^−50^	27.27%	B
	Nam8	TIA1	OMJ95152.1	14%	1 × 10^−7^	35.06%	B
	Snu71	-	N/A	N/A	N/A	N/A	B + T
	Prp42	-	N/A	N/A	N/A	N/A	B + T
	Snu56	-	N/A	N/A	N/A	N/A	B + T
U2 snRNP	Prp21	SF3A1	OMJ90611.1	45%	8 × 10^−40^	36.92%	B
	Prp11	SF3A2	OMJ89506.1	90%	4 × 10^−13^	25.73%	B
	Prp9	SF3A3	OMJ96095.1	75%	4 × 10^−37^	27.65%	B
	Hsh155	SF3B1	OMJ68425.1	84%	0	51.95%	B
	Cus1	SF3B2	OMJ66678.1	63%	2 × 10^−40^	34.05%	B
	Rse1	SF3B3	OMJ94841.1	77%	6 × 10^−50^	26.73%	B
	Hsh49	SF3B4	OMJ96224.1	90%	9 × 10^−38^	37.11%	B
	Ysf3	SF3B5	OMJ86748.1	91%	1 × 10^−29^	53.16%	B
	-	SF3B6	OMJ82020.1	84%	1 × 10^−38^	48.72%	B
	Lea1	SNRPA1	OMJ66761.1	60%	4 × 10^−7^	31.51%	B
	Msl1	SNRPB2	OMJ69199.1	74%	3 × 10^−16^	42.35%	B
	Rds3	PHF5A	OMJ89703.1	98%	4 × 10^−38^	53.33%	B
U2 snRNP associated	Prp5	DDX46	OMJ88772.1	61%	4 × 10^−109^	38.77%	B
	Prp43	DHX15	OMJ88889.1	89%	0	56.81%	B
	Cus2	TATSF1	MPUH01000067.1	29%	3 × 10^−27^	33.33%	T
	Mud2	U2AF2	OMJ92895.1	68%	3 × 10^−41^	30.17%	B
	-	U2AF1	OMJ68977.1	97%	2 × 10^−70^	47.66%	B
	-	CHERP	OMJ90611.1	6%	2 × 10^−7^	48.33%	B
	-	DDX42	OMJ83405.1	46%	2 × 10^−123^	44.75%	B
	-	PUF60	OMJ96224.1	30%	2 × 10^−20^	29.94%	B
	-	U2SURP	N/A	N/A	N/A	N/A	B + T
U5 snRNP	Prp8	PRPF8	MPUH01001036.1	94%	0	59.68%	T
	Brr2	SNRNP200	OMJ93829.1	98%	0	35.16%	B
	Snu114	EFTUD2	OMJ70706.1	99%	5 × 10^−169^	34.24%	B
	Prp6	PRPF6	MPUH01000153.1	54%	5 × 10^−28^	34.15%	T
	Prp28	DDX23	OMJ87031.1	62%	5 × 10^−73^	38.86%	B
	Lin1	CD2BP2	N/A	N/A	N/A	N/A	B + T
	Dib1	TXNL4A	OMJ67094.1	96%	2 × 10^−59^	61.59%	B
	-	SNRNP40	OMJ86718.1	84%	5 × 10^−45^	32.65%	B
U4/U6 snRNP	Prp31	PRPF31	OMJ65893.1	76%	5 × 10-^33^	26.72%	B
	Prp3	PRPF3	OMJ88877.1	46%	7 × 10^−21^	29.91%	B
	Prp4	PRPF4	OMJ68512.1	90%	3 × 10^−38^	27.25%	B
	Snu13	NHP2L1	OMJ79450.1	100%	5 × 10^−54^	61.90%	B
	-	PPIH	OMJ73551.1	94%	4 × 10^−66^	60.36%	B
	-	SART3	OMJ93468.1	18%	2 × 10^−10^	26.29%	B
U4/U6.U5 tri-snRNP	Snu66	SART1	N/A	N/A	N/A	N/A	B + T
	Sad1	USP39	MPUH01000082.1	80%	2 × 10^−21^	24.80%	T
	Hub1	UBL5	OMJ90719.1	98%	2 × 10^−31^	68.06%	B
	Spp381	-	N/A	N/A	N/A	N/A	B + T
RES complex	Bud13	BUD13	OMJ87046.1	24%	1 × 10^−11^	33.12%	B
	Pml1	SNIP1	MPUH01000502.1	81%	3 × 10^−12^	28.74%	T
	Ist3	RBMX2	OMJ73591.1	72%	6 × 10^−34^	54.21%	B
NTC/Prp19 complex	Syf1	XAB2	OMJ71034.1	57%	6 × 10^−30^	23.31%	B
	Clf1	CRNKL1	OMJ94375.1	98%	2 × 10^−111^	33.96%	B
	Cef1	CDC5L	OMJ92975.1	36%	6 × 10^−62^	49.07%	B
	Prp19	PRPF19	OMJ79960.1	61%	2 × 10^−32^	31.09%	B
	Ssa4	HSPA8	OMJ95577.1	94%	0	73.40%	B
	Isy1	ISY1	MPUH01001382.1	53%	9 × 10^−34^	44.44%	T
	Ecm2/Cwc2	RBM22	MPUH01000303.1	70%	6 × 10^−28^	29.46%	T
	Syf2	-	N/A	N/A	N/A	N/A	B + T
	Snt309	SPF27	N/A	N/A	N/A	N/A	B + T
	Ntc20	-	N/A	N/A	N/A	N/A	B + T
	-	PQBP1	N/A	N/A	N/A	N/A	B + T
	-	WBP11	N/A	N/A	N/A	N/A	B + T
	-	CTNNBL1	N/A	N/A	N/A	N/A	B + T
NTC-Related proteins	Prp46	PLRG1	OMJ70863.1	73%	8 × 10^−129^	51.20%	B
	Prp45	SNW1	OMJ67115.1	82%	1 × 10^−18^	28.12%	B
	Bud31	BUD31	OMJ75480.1	100%	1 × 10^−48^	47.13%	B
	-	AQR	OMJ84448.1	34%	3 × 10^−26^	25.33%	B
	-	PPIE	OMJ73551.1	54%	3 × 10^−68^	61.18%	B
	-	PPIL1	OMJ93796.1	87%	9 × 10^−43^	46.90%	B
	Cwc15	CWC15	N/A	N/A	N/A	N/A	B+T
Step 2 proteins	Prp17	PRP17	OMJ92236.1	73%	3 × 10^−70^	36.20%	B
	Prp22	DHX8	OMJ81156.1	73%	0	50.47%	B
	Prp16	DHX38	OMJ82483.1	75%	0	48.62%	B
	Slu7	SLU7	N/A	N/A	N/A	N/A	B + T
	Prp18	PRPF18	N/A	N/A	N/A	N/A	B + T
	Cdc40	CDC40	N/A	N/A	N/A	N/A	B + T
Recruited at A complex	Prp40	PRPF40A	OMJ75909.1	84%	2 × 10^−24^	24.30%	B
	Msl5	SF1	OMJ93203.1	54%	2 × 10^−26^	36.64%	B
	-	RBM5	OMJ90804.1	27%	2 × 10^−8^	26.20%	B
	-	RBM25	OMJ73591.1	72%	6 × 10^−34^	54.21%	B
	-	RBM10	N/A	N/A	N/A	N/A	B + T
	-	CCAR1	N/A	N/A	N/A	N/A	B + T
	-	SUGP1	N/A	N/A	N/A	N/A	B + T
	-	THRAP3	N/A	N/A	N/A	N/A	B + T
Recruited at B complex	Prp38	PRPF38A	OMJ70103.1	55%	1 × 10^−49^	48.84%	B
	Spp382/Ntr1	TFIP11	OMJ89844.1	32%	2 × 10^−32^	35.19%	B
	Snu23	ZMAT2	OMJ87982.1	33%	1 × 10^−10^	39.71%	B
	-	IK	OMJ66867.1	30%	4 × 10^−19^	44.60%	B
	-	MFAP1	OMJ66867.1	55%	1 × 10^−28^	37.75%	B
	-	PRPF4B	OMJ69187.1	36%	3 × 10^−87^	41.24%	B
	-	SMU1	OMJ86718.1	65%	1 × 10^−28^	29.32%	B
	-	WBP4	N/A	N/A	N/A	N/A	B + T
Recruited at B^act^ complex	Cwc22	CWC22	OMJ82911.1	81%	1 × 10^−58^	29.73%	B
	Cwc25	CWC25	OMJ67410.1	38%	6 × 10^−11^	34.73%	B
	Cwc27	CWC27	OMJ68249.1	36%	1 × 10^−67^	58.96%	B
	Prp2	DHX16	OMJ77262.1	75%	0	50.22%	B
	Cwc24	RNF113A	OMJ95226.1	45%	3 × 10^−19^	36.23%	B
	Yju2	YJU2	OMJ95747.1	41%	1 × 10^−21^	38.79%	B
	Spp2	GPKOW	OMJ70620.1	65%	6 × 10^−9^	22.15%	B
	-	CCDC12	OMJ70827.1	48%	3 × 10^−6^	37.04%	B
	-	GPATCH1	OMJ90352.1	7%	5 × 10^−13^	49.30%	B
	-	PPIL2	OMJ93796.1	29%	7 × 10^−37^	45.75%	B
	-	PRCC	N/A	N/A	N/A	N/A	B + T
	-	ZNF830	N/A	N/A	N/A	N/A	B + T
Recruited at C complex	-	CACTIN	OMJ67936.1	27%	2 × 10^−57^	40.82%	B
	-	CDK10	OMJ71086.1	81%	2 × 10^−85^	44.22%	B
	-	DDX41	OMJ89820.1	73%	5 × 10^−117^	39.47%	B
	-	DHX35	OMJ77262.1	91%	0	46.75%	B
	-	FAM32A	OMJ85432.1	44%	6 × 10^−6^	44.00%	B
	-	NOSIP	OMJ75149.1	100%	1 × 10^−20^	24.36%	B
	-	PPIG	OMJ74576.1	22%	3 × 10^−66^	63.10%	B
	-	PPIL3	OMJ93796.1	96%	9 × 10^−49^	51.92%	B
	-	PPWD1	OMJ93796.1	88%	2 × 10^−136^	40.24%	B
	-	SDE2	OMJ76446.1	21%	2 × 10^−14^	34.95%	B
	-	WDR83	OMJ82560.1	87%	4 × 10^−52^	34.29%	B
	-	CXorf56	N/A	N/A	N/A	N/A	B + T
	-	C9orf78	N/A	N/A	N/A	N/A	B + T
	-	DGCR14	N/A	N/A	N/A	N/A	B + T
	-	FAM50A	N/A	N/A	N/A	N/A	B + T
	-	FRA10AC1	N/A	N/A	N/A	N/A	B + T
	-	LENG1	N/A	N/A	N/A	N/A	B + T
hnRNPs	-	HNRNPA1	OMJ89514.1	48%	4 × 10^−40^	41.80%	B
	-	HNRNPAB	OMJ89514.1	49%	4 × 10^−38^	46.07%	B
	-	HNRNPC	OMJ93791.1	23%	2 × 10^−5^	30.99%	B
SR proteins	-	SRSF1-12	N/A	N/A	N/A	N/A	B + T
	-	SREK1	N/A	N/A	N/A	N/A	B + T
	-	SFSWAP	N/A	N/A	N/A	N/A	B + T
	-	TRA2A	N/A	N/A	N/A	N/A	B + T
	-	TRA2B	N/A	N/A	N/A	N/A	B + T
SR related	-	SRRM1	N/A	N/A	N/A	N/A	B + T
	-	SRRM2	N/A	N/A	N/A	N/A	B + T
Others	Pus1	PUS1	OMJ75529.1	52%	2 × 10^−15^	35.94%	B

^a^, ortholog not reported in the species. ^b^ N/A, not applicable due to no significant similarity found by BLASTP and TBLASTN. ^c^ B, BLASTP; T, TBLASTN.

## Supplementary Materials

The following supporting information can be downloaded at: https://www.mdpi.com/article/10.3390/ijms231810973/s1.

## Abbreviations

snRNA	small nuclear RNA
snRNP	small nuclear ribonucleoproteins
SS	splice site
BP-A	branchpoint adenosine

## References

[1] Tartar V. (1961). The Biology of Stentor.

[2] Tartar V. (1963). Extreme alteration of the nucleocytoplasmic ratio in *Stentor coeruleus*. J. Protozool..

[3] Morgan T.H. (1901). Regeneration of proportionate structures in stentor. Biol. Bull..

[4] Slabodnick M.M., Ruby J.G., Reiff S.B., Swart E.C., Gosai S., Prabakaran S., Witkowska E., Larue G.E., Fisher S., Freeman R.M. (2017). The macronuclear genome of stentor coeruleus reveals tiny introns in a giant cell. Curr. Biol..

[5] Sood P., Lin A., Yan C., McGillivary R., Diaz U., Makushok T., Nadkarni A.V., Tang S.K., Marshall W.F. (2022). Modular, cascade-like transcriptional program of regeneration in stentor. eLife.

[6] Grate L., Ares M. (2002). Searching yeast intron data at Ares Lab web site. Methods Enzym..

[7] Hong X., Scofield D.G., Lynch M. (2006). Intron size, abundance, and distribution within untranslated regions of genes. Mol. Biol. Evol..

[8] Hang J., Wan R., Yan C., Shi Y. (2015). Structural basis of pre-MRNA splicing. Science.

[9] Wilkinson M.E., Charenton C., Nagai K. (2020). RNA Splicing by the Spliceosome. Annu. Rev. Biochem..

[10] Will C.L., Luhrmann R. (2011). Spliceosome structure and function. Cold Spring Harb. Perspect. Biol..

[11] Fica S.M., Nagai K. (2017). Cryo-electron microscopy snapshots of the spliceosome: Structural insights into a dynamic ribonucleoprotein machine. Nat. Struct. Mol. Biol..

[12] Chanarat S. (2021). UBL5/Hub1: An atypical ubiquitin-like protein with a typical role as a stress-responsive regulator. Int. J. Mol. Sci..

[13] Galej W.P., Wilkinson M.E., Fica S.M., Oubridge C., Newman A.J., Nagai K. (2016). Cryo-EM structure of the spliceosome immediately after branching. Nature.

[14] Cordin O., Beggs J.D. (2013). RNA helicases in splicing. RNA Biol..

[15] Cordin O., Hahn D., Beggs J.D. (2012). Structure, function and regulation of spliceosomal RNA helicases. Curr. Opin. Cell Biol..

[16] StentorDB Stentor Genome Database Wiki. http://stentor.ciliate.org/index.php/home/welcome.

[17] Crooks G.E. (2004). WebLogo: A sequence logo generator. Genome Res..

[18] McCoy M.J., Fire A.Z. (2020). Intron and gene size expansion during nervous system evolution. BMC Genom..

[19] Sakurai A., Fujimori S., Kochiwa H., Kitamura-Abe S., Washio T., Saito R., Carninci P., Hayashizaki Y., Tomita M. (2002). On biased distribution of introns in various eukaryotes. Gene.

[20] Griffiths-Jones S., Bateman A., Marshall M., Khanna A., Eddy S.R. (2003). Rfam: An RNA family database. Nucleic Acids Res..

[21] Infernal 1.1: 100-Fold Faster RNA Homology Searches Bioinformatics Oxford Academic. https://academic.oup.com/bioinformatics/article/29/22/2933/316439?login=false.

[22] De Maio A., Yalamanchili H.K., Adamski C.J., Gennarino V.A., Liu Z., Qin J., Jung S.Y., Richman R., Orr H., Zoghbi H.Y. (2018). RBM17 interacts with U2SURP and CHERP to regulate expression and splicing of RNA-processing proteins. Cell Rep..

[23] Chanarat S., Sträßer K. (2013). Splicing and beyond: The many faces of the Prp19 complex. Biochim. Biophys. Acta (BBA) Mol. Cell Res..

[24] Pastor F., Shkreta L., Chabot B., Durantel D., Salvetti A. (2021). Interplay between CMGC kinases targeting SR proteins and viral replication: Splicing and beyond. Front. Microbiol..

[25] Fukuhara T., Hosoya T., Shimizu S., Sumi K., Oshiro T., Yoshinaka Y., Suzuki M., Yamamoto N., Herzenberg L.A., Herzenberg L.A. (2006). Utilization of host SR protein kinases and RNA-splicing machinery during viral replication. Proc. Natl. Acad. Sci. USA.

[26] Wagner R.E., Frye M. (2021). Noncanonical functions of the serine-arginine-rich splicing factor (SR) family of proteins in development and disease. BioEssays.

[27] Liao S.E., Regev O. (2021). Splicing at the phase-separated nuclear speckle interface: A model. Nucleic Acids Res..

[28] Twyffels L., Gueydan C., Kruys V. (2011). Shuttling SR proteins: More than splicing factors. FEBS J..

[29] Henry M.F., Mandel D., Routson V., Henry P.A. (2003). The yeast HnRNP-like protein Hrp1/Nab4 accumulates in the cytoplasm after hyperosmotic stress: A novel Fps1-dependent response. MBoC.

[30] Loya T.J., O’Rourke T.W., Reines D. (2013). Yeast Nab3 protein contains a self-assembly domain found in human heterogeneous nuclear Ribonucleoprotein-C (HnRNP-C) that is necessary for transcription termination. J. Biol. Chem..

[31] The HnRNP Family: Insights into Their Role in Health and Disease SpringerLink. https://link.springer.com/article/10.1007/s00439-016-1683-5.

[32] Zenklusen D., Vinciguerra P., Strahm Y., Stutz F. (2001). The yeast HnRNP-like proteins Yra1p and Yra2p participate in MRNA export through interaction with Mex67p. Mol. Cell. Biol..

[33] Sheng J., Gan J., Soares A.S., Salon J., Huang Z. (2013). Structural insights of non-canonical U•U pair and Hoogsteen interaction probed with Se atom. Nucleic Acids Res..

[34] Galaxy Platform for Accessible, Reproducible and Collaborative Biomedical Analyses: 2018 Update Nucleic Acids Research Oxford Academic. https://academic.oup.com/nar/article/46/W1/W537/5001157.

[35] (2015). RStudio: Integrated Development for R. http://www.rstudio.com.

[36] Raudvere U., Kolberg L., Kuzmin I., Arak T., Adler P., Peterson H., Vilo J. (2019). G:Profiler: A web server for functional enrichment analysis and conversions of gene lists (2019 Update). Nucleic Acids Res..

[37] CLUSTAL W: Improving the Sensitivity of Progressive Multiple Sequence Alignment through Sequence Weighting, Position-Specific Gap Penalties and Weight Matrix Choice Nucleic Acids Research Oxford Academic. https://academic.oup.com/nar/article-abstract/22/22/4673/2400290?redirectedFrom=fulltext&login=false.

[38] pyBoxshade/BS_app.py at master · mdbaron42/pyBoxshade · GitHub. https://github.com/mdbaron42/pyBox-shade.

[39] GraphPad Prism 9 User Guide—How to Cite GraphPad Prism. https://www.graphpad.com/guides/prism/latest/user-guide/citing_graphpad_prism.htm.

[40] Bateman A., Martin M.-J., Orchard S., Magrane M., Agivetova R., Ahmad S., Alpi E., Bowler-Barnett E.H., Britto R., The UniProt Consortium (2021). UniProt: The universal protein knowledgebase in 2021. Nucleic Acids Res..

[41] BLAST: At the Core of a Powerful and Diverse Set of Sequence Analysis Tools Nucleic Acids Research Oxford Academic. https://academic.oup.com/nar/article/32/suppl_2/W20/1040657?login=false.

[42] Zimmermann L., Stephens A., Nam S.-Z., Rau D., Kübler J., Lozajic M., Gabler F., Söding J., Lupas A.N., Alva V. (2018). A completely reimplemented MPI bioinformatics toolkit with a new HHpred server at its core. J. Mol. Biol..

[43] Šali A., Blundell T.L. (1993). Comparative protein modelling by satisfaction of spatial restraints. J. Mol. Biol..

[44] Pettersen E.F., Goddard T.D., Huang C.C., Meng E.C., Couch G.S., Croll T.I., Morris J.H., Ferrin T.E. (2021). UCSF ChimeraX: Structure visualization for researchers, educators, and developers. Protein Sci..

