# Supplementary material for: Insights into the Mechanism of Pre-mRNA Splicing of Tiny Introns from the Genome of a Giant Ciliate Stentor coeruleus
Source: Int J Mol Sci. 2022 Sep 19;23(18):10973. doi: 10.3390/ijms231810973 (PMC9505925; doi:10.3390/ijms231810973)
Supplement: Supplementary file 1 [file ijms-23-10973-s001.zip › ijms-1883101-supplementary-resubmit-1.pdf]

Supplementary data

Insights into the mechanism of pre-mRNA splicing of tiny introns from the genome of a giant ciliate *Stentor coeruleus*

Jirayu Nuadthaisong, Tanaporn Phetruen[#], Chanakan Techawisutthinan[#], Sittinan Chanarat[*]

Laboratory of Molecular Cell Biology, Department of Biochemistry, Faculty of Science, Mahidol University, Bangkok, 10400, Thailand.

# These authors contributed equally to this work

* Corresponding author: sittinan.cha@mahidol.ac.th

Supplementary figure legends

Figure S1. Functional enrichment analysis of genes harboring introns in *S. coeruleus.* Gene ontology analysis was performed on g:Profiler using *Tetrahymena thermophila* (A) and *Paramecium tetraurelia* (B), two closely related ciliates in phylum Ciloiphora, as organism input parameter. GO, Gene ontology; MF, molecular function; BP, biological process; CC, cellular component; KEGG, Kyoto Encyclopedia of Genes and Genomes pathway. Padj, a p-value adjusted for multiple testing using the Benjamini-Hochberg method [1].

Figure S2. Annotated tiny introns of S. coeruleus are functional and indeed spliced out. Comparison between sequences of four different genes harboring introns at the genomic DNA and mRNA level. Genomic DNA sequences were retrieved from the StentorDB [2], the mRNA sequences from RNAseq experiment [3]. Intronic regions are highlighted in red. Amino acid sequences translated corresponding mRNA are also shown below each alignment pair. Note that, for simplicity purpose, U at the mRNA level is shown as T. (A) SteCoe_2110, (B) SteCoe_13333, (C) SteCoe_32708, and (D) SteCoe_38208 genes were analyzed.

Figure S3. Results from 'cmsearch' program in the Infernal package (A) Alignment from cmsearch result represents matching between U1 snRNA candidate from *S. coeruleus* and covariance model (CM) of eukaryotic U1 snRNA (Rfam: RF00003). (B) – (E) Similar to Fig. S1A, but CMs of U2 (Rfam: RF00004), U4 (Rfam: RF00015), U5 (Rfam: RF00020), and U6 snRNAs (Rfam: RF00026) were used as queries. Contigs and genomic locations of the *S. coeruleus* U-snRNA candidates are also indicated.

Figure S4. A simplified proposed model of RNA interaction network during pre-B complex

Figure S5. Length of predicted spliceosomal proteins of Stentor versus human proteins Comparison of the predicted proteins from S. coeruleus versus their human protein homologs. The line of identity (1:1) is shown as dashed line. aa: amino acids.

Figure S6. Certain parts of the branching factors that are adjacent to the spliceosome active site are noticeably non-conserved. Sequence alignments of the three branching factors Yju2/CCDC94 (A), Cwc25/CCDC49 (B), and Ntc30/ISY1 (C) are shown. N-terminal regions of proteins that are projected to the spliceosomal active site are enclosed in red dashed boxes.

Table S1. Functional enrichment analysis of genes harboring introns in *S. coeruleus.* Similar to Figure S1, but the full set of results were shown in table.

References

1. Raudvere, U.; Kolberg, L.; Kuzmin, I.; Arak, T.; Adler, P.; Peterson, H.; Vilo, J. G:Profiler: A Web Server for Functional Enrichment Analysis and Conversions of Gene Lists (2019 Update). *Nucleic Acids Research* **2019**, *47*, W191–W198, doi:10.1093/nar/gkz369.
2. StentorDB | Stentor Genome Database Wiki Available online: http://stentor.ciliate.org/index.php/home/welcome.
3. Sood, P.; Lin, A.; Yan, C.; McGillivary, R.; Diaz, U.; Makushok, T.; Nadkarni, A.V.; Tang, S.K.; Marshall, W.F. Modular, Cascade-like Transcriptional Program of Regeneration in Stentor. *eLife* **2022**, *11*, e80778, doi:10.7554/eLife.80778.

Figure S1

A

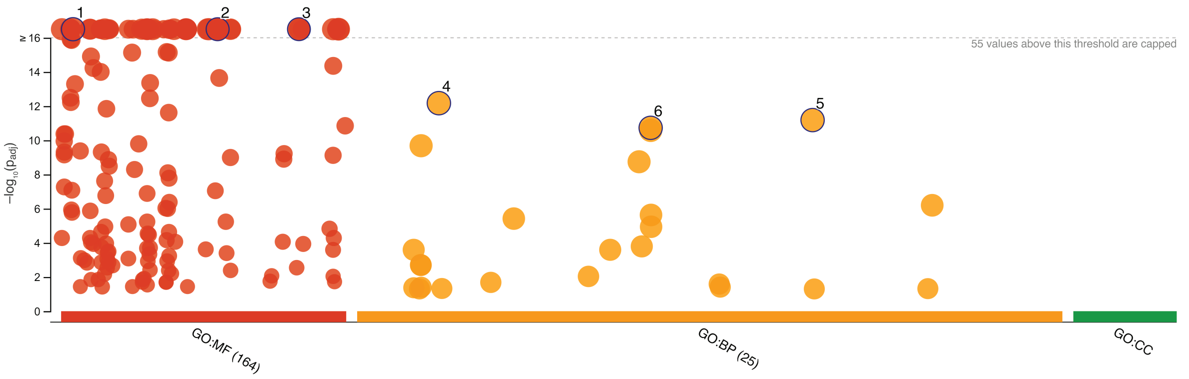

ID	Source	Term ID	Term Name	p_{adj} (query_1)
1	GO:MF	GO:0003824	catalytic activity	4.941×10^{-324}
2	GO:MF	GO:0043167	ion binding	4.941×10^{-324}
3	GO:MF	GO:0097159	organic cyclic compound binding	2.283×10^{-303}
4	GO:BP	GO:0008152	metabolic process	6.724×10^{-13}
5	GO:BP	GO:0071704	organic substance metabolic process	6.572×10^{-12}
6	GO:BP	GO:0044237	cellular metabolic process	1.863×10^{-11}

B

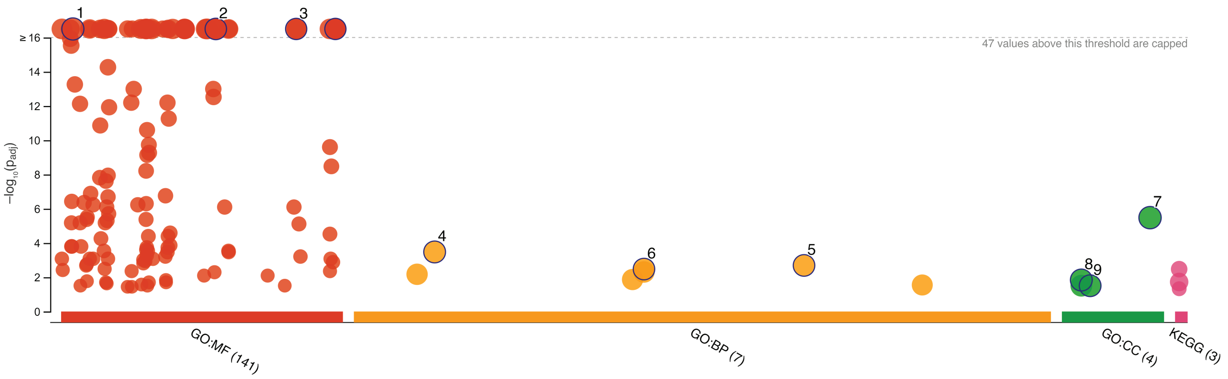

ID	Source	Term ID	Term Name	p_{adj} (query_1)
1	GO:MF	GO:0003824	catalytic activity	4.941×10^{-324}
2	GO:MF	GO:0043167	ion binding	3.324×10^{-292}
3	GO:MF	GO:0097159	organic cyclic compound binding	1.395×10^{-253}
4	GO:BP	GO:0008152	metabolic process	3.328×10^{-4}
5	GO:BP	GO:0071704	organic substance metabolic process	2.037×10^{-3}
6	GO:BP	GO:0044238	primary metabolic process	3.316×10^{-3}
7	GO:CC	GO:0110165	cellular anatomical entity	3.305×10^{-6}
8	GO:CC	GO:0016020	membrane	1.449×10^{-2}
9	GO:CC	GO:0031224	intrinsic component of membrane	3.115×10^{-2}

Figure S2

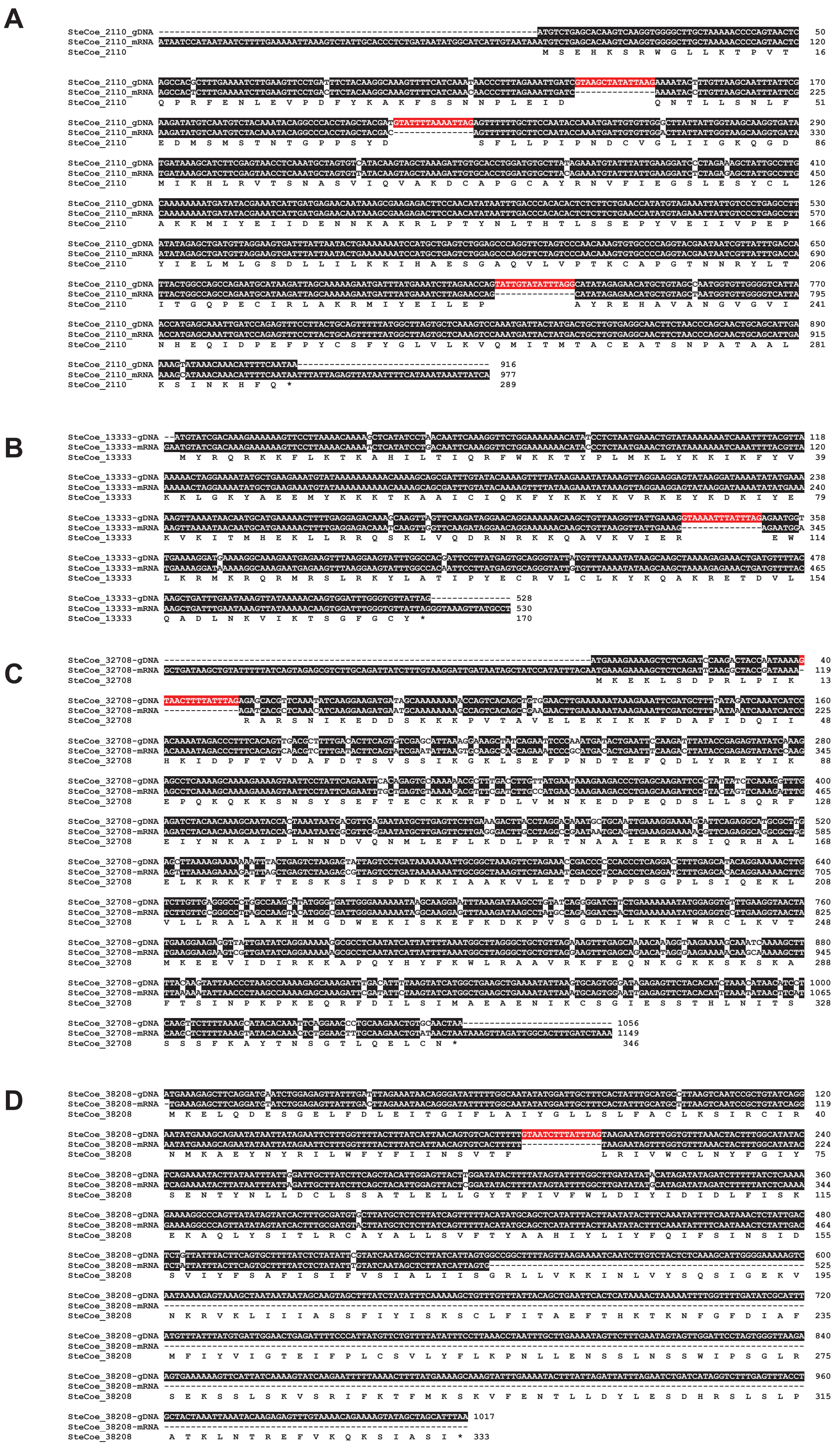

Figure S3

A

```
                                    v      v                    v                                              NC
                       :::::::::::((((<<<<<<-<<<<<__________>>>>>>>>>>>,<<<------<<<-<<___._______>>-->>->----->> CS
             U1      1 AUACUUACCUGGccggGGggaaacggcGAUCAaGAAGgccguucccCCcagGgugaGGucccucCAUU.GCACUucGgagggugcugaccC 89
                         ACUUACCUGG  G::G:G  :C:::GAUCAAGAAG:::G: C:C::AGGG ++G +C:: :CAUU GCACUU+G:+G:: G+  A CC
SteCoe_contig_324 9049 UCACUUACCUGGUUGGAGUGUGUCUUUGAUCAAGAAGGAAGACCGCUUAGGG-UGGAGCAGGUCAUUcGCACUUUGAUGCU-GUAAAGCC 8962
                       ***********************************************8888.788889999*****************98.89999999 PP

                                                      v                          v      v           NC
                       >,,<<<-<<-<<______>>->>--->>>,))))--------------<<<<<--<<<<<____>>>>>->>>>>: CS
             U1     90 cuGcGgUuccCCCaAAGUGGugaaAccCgAcggCAUAAUUUgUGgUAGucgGGGgaccGcgUUcGcgCggcCCCcgc 166
                       CU C:G U: CCC     GG :A A C:GAC  C  AAUUU UGG AGU:GGG: +::GC  U+G  C::C:CCC:
SteCoe_contig_324 8961 CUACAGCUU-CCCGU-UCGG-GACAGCUGACUGCUAAAUUUCUGGCAGUGGGGA-CGUGCGCUUGU-CGCCUCCCCG 8890
                       999****99.77777.7999.888*****************************.8999999875.8899****** PP
```

B

```
                                                                                                  v   v            NC
                        ::::::<<<-<<<<____>>>>->>>,,,,,,,,,,,,,,,,,,,,<<<<<<________>>>>>>,<<<<<<<___>>>>>>>,,,, CS
              U2      1 AUacCUUCucgGCcUUUUgGCuaaGAUCAAGUGUAGUAUCUGUUCUUauCAGUuUAAuAuCUGauAuggcccccAuuggggccaauu 88
                        AUACCUUCUCGGC UUUU GCUAAGAUCAAGUGUAGUAUCUGUUCUUA:CAGU+U A A+CUG:UAU:::C:C A   G:G:::   U
SteCoe_contig_507 11317 AUACCUUCUCGGCUUUUUGGCUAAGAUCAAGUGUAGUAUCUGUUCUUAUCAGUGUGAAAACUGGUAUCCACUCUAC-UGAGUGGCG-U 11402
                        *****************************************************************************.*********.* PP

                                                v      v                  v      v         v               NC
                        ,,,,,,,,,,,,,,,,,,,,,,,,<<<<<<<<----<<<<<__>>>>>-->>>>>>>>,,<<<<<<-<<<<<<__________._>>> CS
              U2     89 auaUUAaauuaAUUUUUggaacuaGugggggcauuuuggGCUUGCccauugcccccaCacggguugaccuggcaUUGCAcUac.cgcc 175
                        +U+U A AUUAAUUU U GA  +  ::::GG  UUU:::GCUUGC::: + CC:::: A+ GGU:G  CU:G:AUUGCACU+  C:C:
SteCoe_contig_507 11403 UUUUCACAUUAAUUUCUUGACGGGAGCGAGGCCUUUCCAGCUUGCUGGGAACCUUGCAAUGGGUCGCCCUUGUAUUGCACUUAcCUCA 11490
                        ********************************************************************************98889*** PP

                          v               NC
                        >>>-->>>>>>::::::: CS
              U2    176 agguucagcccAcccuuu 193
                        AG   C:GCC ACCCU++
SteCoe_contig_507 11491 AGUGGCGGCCUACCCUCC 11508
                        ****************** PP
```

C

```
                                                                                                      v  NC
                        ::::::::::.::::::<<-<<<<<-<<<_________________>>>>>>>>-->>,,,,<<<<____________>>>>,,,,<<< CS
              U4      1 AUCcUUGuGC.AgGGGcAaUaccccugcCAGUGAGGaUucguCcGAGgcggggUauUGCUAgUugAAAACUAuuucccaAcaccCgcc 88
                        A CCUUGUGC AGGGGCAAUA:C:C+GC:AGUGA G UU+  C+GA:GCG:G:U AUUGCUA:UU:AAAACUA+U CC:AA:   CG+
SteCoe_contig_1964 3831 AGCCUUGUGCuAGGGGCAAUAUCGCAGCAAGUGAAGCUUA-GCUGAUGCGCGAUGAUUGCUAGUUGAAAACUAAU-CCAAAU---CGU- 3749
                        ****************************************.***********************************.989999...688. PP

                                                  v                         NC
                        <<<<<<<<<<____>>>>>>>>>>->>>,,,,,,,,,,,,<<<<____>>>> CS
              U4     89 ucauggccucuucagaggccaugaaggcAAUUUUUGgAAuccccuuaagggg 140
                         :::::::    C   :::::::    CAAUUU UGG A :::C +AAG:::
SteCoe_contig_1964 3748 GGUCGUUU--ACCC--AAAUGACU---CAAUUUCUGGCA-GGUCGAAAGACC 3705
                        77888887..8888..78888877...9*********99.************ PP
```

D

```
                                                                                                           NC
                        ::<<<<--<<<<<<<<<<<-------<<<<<<<___________>>>>>>>>--->>>>>>>>>>>------>>>>----------- CS
              U5      1 auccccuGgggccacuacauauCGAAcaagucUcUcGCCUUUUACuAgAgacuuCCGuguaguggcccaauuaaggguuuaaacuAAU 89
                        + :C:C GG ::CA:U:CA+A+CGA +:A:UCU:UCGCCUUUUACUA:AGA:U:CCGUG:A:UG:: C    ++G:G:UU AA C A U
SteCoe_contig_1638 8263 UAUCGCAGGGUUCAGUUCAAAACGAUUUAAUCUUUCGCCUUUUACUAAAGAUUACCGUGAACUGGGUCUUAGUUGCGAUUGAACC-AUU 8350
                        ************************************************************************************99.*** PP

                              v                   v NC
                        ------<-<<<<<<______>>>>>>> CS
              U5     90 UUUUGgaagcccuuuuuuuuaagggcu 116
                        UUUUG   :::CUU      AAG:::
SteCoe_contig_1638 8351 UUUUGCAGCGGCUUA-----AAGCCGC 8372
                        *******88888885.....8888888 PP
```

E

```
                          v          v                                                                             NC
                        <<<<<______>>>>>::::::::::::::::::::::::::::::::::::::::::::::::::::::::::::::::.::::: CS
              U6      1 guccccUUCGggggacaUaUggUaAAaUUGgAACgAUACAGAGAAGAUUAGCAUGGCCCCUGCaCAAGGAUGACACguauaau.agAGA 88
                        G: CCCUU   GG :CAU  G+U AAAUUGGAACGAUACAGAGAAGAUUAGCAUGGCCCCUGC+CAAGGAUGACACG++ AA+ +GAGA
SteCoe_contig_1373 7452 GAGCCCUUU--GGGUCAUCCGAUUAAAUUGGAACGAUACAGAGAAGAUUAGCAUGGCCCCUGCGCAAGGAUGACACGCUCAAAuCGAGA 7366
                        *****6665..6669*************************************************************************** PP

                                        NC
                        ::::::::::::::: CS
              U6     89 gauguaccauaUUUUU 104
                        ++UGUA C  A  UUU
SteCoe_contig_1373 7365 AGUGUAUCCCAAAUUU 7350
                        **************** PP
```

Figure S4

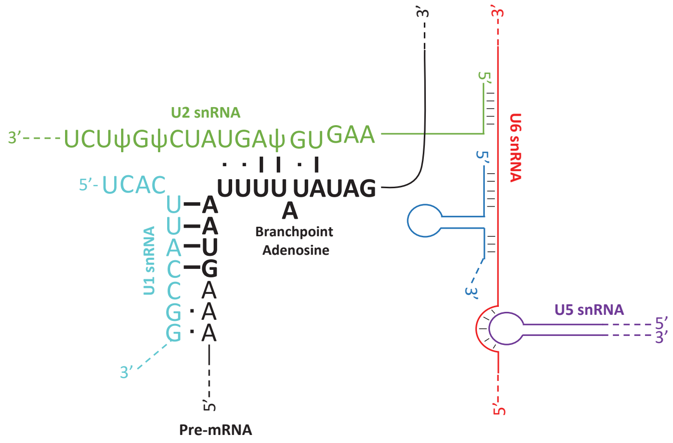

U2 snRNA
3′----UCUψGψCUAUGAψGUGAA
5′-UCAC
UUUU UAUAG
A
Branchpoint
Adenosine
U1 snRNA
U A U G A A A
Pre-mRNA
U6 snRNA
U5 snRNA

Figure S5

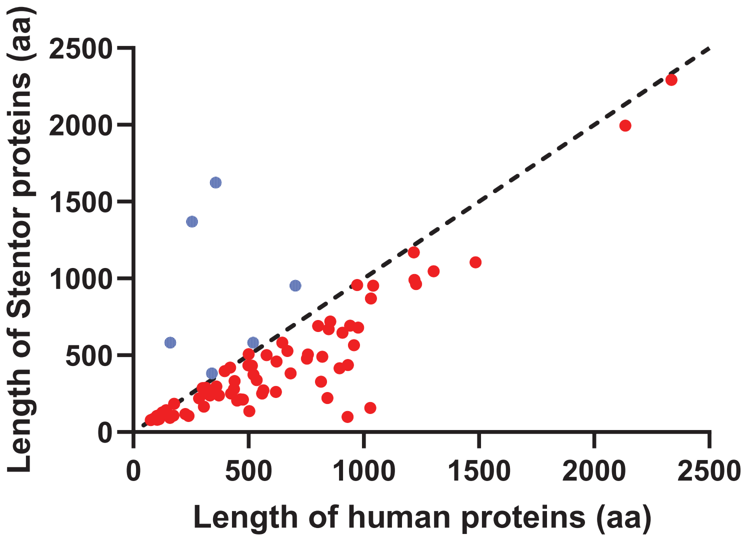

Length of Stentor proteins (aa)
2500
2000
1500
1000
500
0
0
500
1000
1500
2000
2500
Length of human proteins (aa)

Figure S6

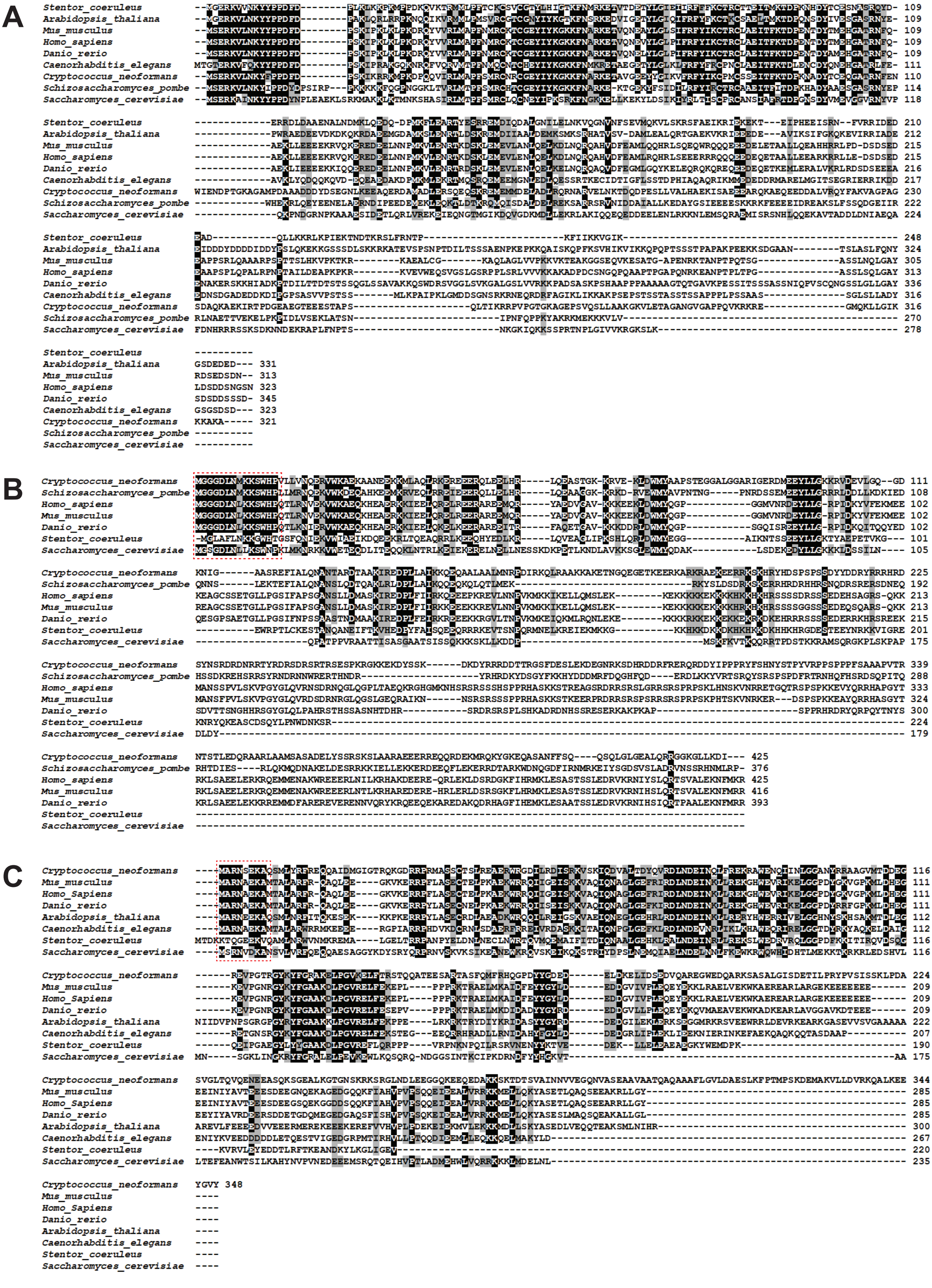
A
B
C
Stentor_coeruleus
Arabidopsis_thaliana
Mus_musculus
Homo_sapiens
Danio_rerio
Caenorhabditis_elegans
Cryptococcus_neoformans
Schizosaccharomyces_pombe
Saccharomyces_cerevisiae
